# Occupational Pain Medicine: From Paradigm Shift in Pain Neuroscience to Contextual Model of Care

**DOI:** 10.3389/fnhum.2019.00188

**Published:** 2019-06-06

**Authors:** Steven M. Miller

**Affiliations:** ^1^Perceptual and Clinical Neuroscience Laboratory, Department of Physiology, Monash Biomedicine Discovery Institute, School of Biomedical Sciences, Monash University, Melbourne, VIC, Australia; ^2^Monash Alfred Psychiatry Research Centre, Central Clinical School, Monash University and Alfred Health, Melbourne, VIC, Australia

**Keywords:** occupational pain medicine, models of care, pain neuroscience, work disability, persistent pain, biopsychosocial model, pain management, compensation

## Overview

Persistent low back pain and neck pain are major causes of years lived with disability in industrialized and developing countries (Vos et al., 2015). These and other musculoskeletal and neuropathic persistent pain (PP) conditions cause enormous personal, social, and economic burden of illness and present major health management and health economic challenges. Such challenges may be amplified if the cause of an injury or aggravation of a condition is deemed compensable, and thus managed within the context of compensation schemes (which are often cumbersome). In the last two decades the neuroscience of pain has undergone a paradigm shift in which the role of higher-level, cortical neuronal processes—driven by a multitude of contextual factors relevant to the individual experiencing pain—has emerged as a key driver of PP. This knowledge paradigm shift has been accompanied by a more gradual, indeed lagging, implementation shift in PP management. There is now a widening, though far from universal, recognition of the value of reducing emphasis on mechanical pathoanatomical biomedical models of care (MoCs) in favor of contextual biopsychosocial MoCs (e.g., the Australian-based “Choosing Wisely” guidelines, the UK-based “NICE” guidelines and recommendations in a recent *Lancet* special issue on low back pain; Vol. 391, No. 10137, March 21, 2018).

MoCs are evidence-based policies or frameworks that recommend how healthcare is best provided to consumers and factor in local operational requirements (Briggs et al., 2014). Beales et al. (2016) recently published detailed analyses of helpful and unhelpful MoCs for PP management in a compensable environment (see below). Some authors of that publication are indeed promoting an education-based “Pain Revolution” (https://www.painrevolution.org), the goal of which is to rethink the way pain is discussed and managed, so as to fully engage with the pain neuroscience paradigm shift. Beales et al. (2016) proposed an integrated MoC to optimize the journey of an injured worker through the compensation environment, focusing on two overarching principles: (i) application of a biopsychosocial approach incorporating contemporary pain neuroscience; and (ii) recognizing that (good) work is good for your health.

Here I sketch key features of what might be deemed a species of the MoC outlined by Beales et al. (2016). Termed “Occupational Pain Medicine” (OPM; or “Occupational Pain Management”—see below), the MoC is firmly founded on the analyses, recommendations, terminology, and practice points outlined in Beales et al. (2016). By its very name though, the OPM MoC underscores their two overarching principles, and importantly, suggests further specific implementation strategies. At the core of the OPM MoC is the *formulation* at which the treating practitioner or medicolegal assessor should arrive after clinical assessment of the individual and detailed consideration of their general and occupational context.

## The Paradigm Shift in Pain Neuroscience

Biopsychosocial approaches to managing PP target dysfunctional cognitions, behaviors and other psychosocial drivers (Loeser, 1982; Gatchel et al., 2007). It has been argued however, that their goal of enabling individuals with PP to live well *with* their pain may not be optimal (Moseley and Butler, 2015). Rather, it has been proposed that the bolder aim of living well *without* pain can be achieved by educating individuals with PP about principles of contemporary pain neuroscience (Moseley, 2003, 2007; Moseley et al., 2004; Moseley and Butler, 2015)^1^. The “Explain Pain” (EP) program and educational materials [reviewed in Moseley and Butler (2015) including its evidence base] have as their core objective, belief shifting. The conceptualization of pain is proposed to shift from a marker of tissue damage or disease to instead the perceived need to protect bodily tissue. This reconceptualises pain itself rather than pain-related disability. EP conveys that pain increases with evidence of danger to bodily tissues and conversely, decreases with evidence of safety. The following are key EP principles for individuals with PP to appreciate (Moseley and Butler, 2015): “the variable relationship between danger messages (nociception) and pain; the potent influence of context on pain; upregulation in the danger transmission (nociceptive) system as pain persists; the coexistence of several potential protective systems, of which pain is one, but the only one that the sufferer necessarily knows has been engaged; the potential influence of these other protective systems on pain; the adaptability, and therefore trainability, of our biology (including but not limited to the concept of neuroplasticity) and the knowledge that this adaptation back to normality is likely to be slow.”

In understanding these principles, the individual with PP becomes pain literate and understands (in contemporary terms) how pain is produced, maintained, and modulated. This knowledge is then integrated into perceptions and beliefs about pain and function, and consequently attitudes, behaviors, treatments, and lifestyle choices (Moseley and Butler, 2015). A range of additional principles are relevant to the paradigm shift that has occurred in contemporary pain neuroscience and these, along with pain literacy fundamentals, are presented in detail in Beales et al. (2016). Two worth mentioning include: (i) limitations inherent in structurally-focused pathoanatomical biomedical management approaches (discussed below); and (ii) powerful context-related modulating factors of placebo and nocebo effects in clinical interactions (Arnold et al., 2014). Placebo effects, though potentially helpful, can negatively impact the choice and repeating of biomedical interventions, and nocebo effects can increase pain through danger messages conveyed by practitioners (e.g., “you have the back of a 90-year old”).

The paradigm shift in pain neuroscience described above draws on fundamental pain literacy concepts such as nociception, sensitization, and neuroplasticity, and importantly, the recognition that cortical neuronal processes modulate ascending nociceptive input to pain. While precise mechanisms remain to be determined, two relevant cortical regions are anterior cingulate cortex (ACC) and anterior insular cortex (AIC; Rainville et al., 1997; Apkarian et al., 2005; Ploner et al., 2010; Wiech et al., 2010). Indeed, these regions are relevant not only to processing pain but also salience (hence salience of pain to the individual) and are additionally implicated in the pathophysiology of almost all psychiatric disorders (Downar et al., 2016; Miller, 2016). Moreover, in an important recent pain neuroscience development, a rodent study published in *Nature* reports that allodynia—pain from a usually non-painful stimulus and a key marker of neuropathic pain and sensitized pain pathways—is modulated by descending corticospinal tract (CST) neurones previously thought to mediate only motor function (Liu et al., 2018). This suggests a novel top-down mechanism by which cortical structures and contextual factors might modulate PP. Relevant to further discussion below, some of the allodynia-modulating CST neurones originate in the secondary somatosensory area (S2).

## Occupational Pain Medicine: A Model of Care for Preventing and Managing Persistent Pain

As mentioned above, the OPM MoC can be considered a species of the integrated MoC proposed by Beales et al. (2016). Those authors reviewed helpful and unhelpful aspects of PP management in compensation environments. A detailed overview of their analyses and recommendations is beyond the scope of this Opinion article, but the present proposal for an OPM MoC should be read in conjunction with their article. Briefly, Beales et al. (2016) examined the Australian workers compensation system (with relevance to some international schemes) and criticized the unhelpful ongoing dominance of biomedical constructs at all levels of the scheme. Biomedical constructs hold that a structural or pathoanatomical anomaly (or “pain generator”) causes PP and that if “fixed,” pain will be eliminated or reduced. In practice however, surgical “fixing” of PP is notoriously unhelpful (with exceptions, such as hip or knee replacement). Indeed, failing to appreciate and deal with the context in which PP arises and is propagated is likely to be met with multiple treatment failures and thus reinforced negative contextual drivers (Beales et al., 2016).

Beales et al. (2016) also review helpful and unhelpful occupational and insurance contexts within which work injuries are managed, the critical role of and strategies for achieving a timely and sustainable return to work (RTW), and implementation strategies for improving RTW (e.g., Buchbinder et al., 2001; Loisel et al., 2001; Franche et al., 2005, 2009; Waddell and Burton, 2005; McCluskey et al., 2006; Schultz et al., 2007; Damschroder et al., 2009; Carroll et al., 2010; Pransky et al., 2011; Caruso, 2013; Aurbach, 2014; van Vilsteren et al., 2015; Linton et al., 2016). They argue that optimizing compensation environments enables positive helpful injury journeys for individuals and thus improved outcomes, and can be achieved by recognizing the two overarching principles mentioned above and integrating such recognition at all levels: system (legislation, structure and policy), organizational (regulatory bodies, insurers, employers, workplaces), and individual (individuals with PP, families, practitioners, supervisors, co-workers, medicolegal assessors, clinical panels). Inter- and intra-level stakeholder communication is emphasized, as is requirement for active participation of the injured individual.

**Figure 1:**
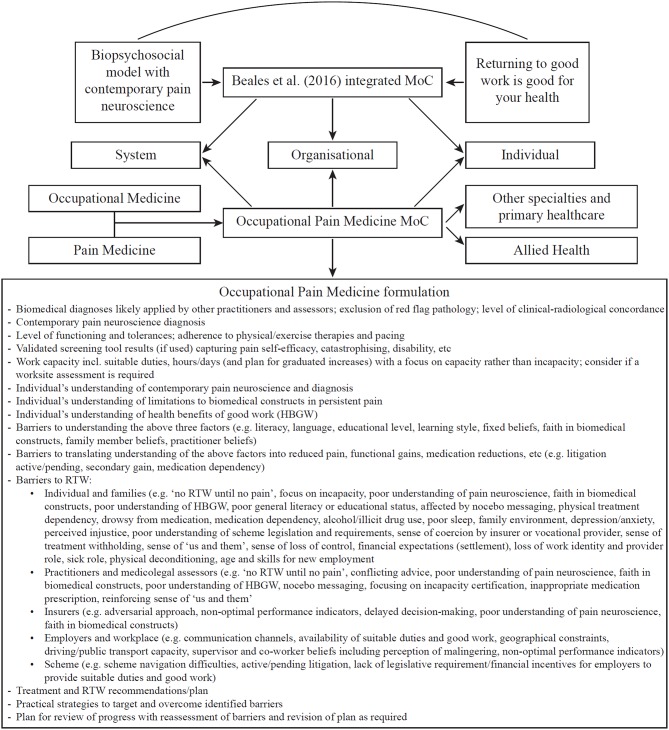
The OPM MoC consists of three aspects. First, its foundations are the analyses, recommendations, terminology, and practice points already laid out in detail by Beales et al. (2016). Second, the OPM MoC by its very name encourages Occupational Medicine to better appreciate and implement contemporary pain neuroscience and Pain Medicine to better appreciate and implement RTW. In addition to thus remedying deficiencies in each of these medical specialties' approach to managing PP, the OPM MoC yields further strategies to integrate the two overarching principles at all three levels of compensation schemes. For example, one new strategy is the garnering of influential faculty support and lobbying for such integration from the Australasian Faculty of Occupational and Environmental Medicine and the Australian Faculty of Pain Medicine. These peak bodies can influence system-wide and legislative changes, practitioner remuneration changes, and medical curriculum changes (at undergraduate, post-graduate, and continuing education levels), all geared toward an optimized MoC for managing PP. Third, a core feature of the OPM MoC and a further recommended strategy for optimizing the compensation journey for the person with PP, is the formulation at which the treating practitioner or medicolegal assessor should arrive following clinical assessment of the individual and detailed consideration of their general and occupational context. A non-exhaustive list of elements for the OPM formulation is provided.

With this background, I propose the OPM MoC to provide further multilevel strategies to optimize the injured individual's compensation journey (detailed in Figure 1). The recommendations can also be extrapolated to non-compensable environments, with some limitations (such as the lack of legislative requirement for employers to provide suitable duties for non-compensable individuals). The stimulus for proposing the OPM MoC is this author's experience over many years managing injured individuals and providing clinical panel advice to both workers and transport accident compensation schemes (involving review of thousands of compensable cases). This experience has led to the view that, in Australia at least, Occupational Medicine appreciates and implements RTW well, but not contemporary pain neuroscience, while Pain Medicine appreciates and implements contemporary pain neuroscience well (at least in some settings)^2^, but not RTW. The clinical panel role also provides unique perspective on the great variability with which PP is managed within and across disparate medical specialties (Occupational Medicine, Pain Medicine, Neurology, Rheumatology, Orthopedic Surgery, Neurosurgery, Musculoskeletal Medicine, Sport and Exercise Medicine, Rehabilitation Medicine, etc). From this vantage point it is evident that: (i) biomedical constructs continue to prevail; (ii) biopsychosocial approaches are implemented only to some extent; and (iii) contemporary pain neuroscience is generally poorly appreciated. The OPM MoC aims to synthesize what Occupational Medicine and (contemporary) Pain Medicine do well in managing PP, thus remedying deficiencies in each. It also provides a heuristic for any medical field that manages PP. Although the model primarily targets medical practitioners, it can nonetheless be usefully adopted by allied health practitioners (e.g., physiotherapists, occupational therapists, rehabilitation providers) by exchanging “Medicine” in OPM to “Management.” Indeed, reflecting a multidisciplinary approach, pain programs that utilize the OPM MoC might be best entitled “Occupational Pain Management Programs.”

Finally, it is worth commenting on the role of surgical and interventional pain management procedures within the OPM MoC. These are appropriate for red flag or urgent pathologies (e.g., surgery for cauda equina syndrome) and may be justifiable for some structural anomalies if: (i) there are clearly concordant clinical-radiological findings; (ii) there is high-level evidence supporting the procedure; (iii) the individual is fully and accurately informed about the procedure's chances of success and possible complications; and (iv) there has been opportunity for (contemporary) conservative PP management. In practice however, PP cases are seldom clinically-radiologically concordant and the evidence base for common PP management procedures is often low-level or worse. Nonetheless, if best efforts at pain education without nocebo messaging have failed, then reversible interventions with a trial phase—such as neuromodulation with spinal cord stimulation, which has a growing evidence base (Verrills et al., 2016) though has yet to be adequately assessed by sham-controlled trials to control for placebo effects—are preferred to major irreversible interventions such as spinal fusion surgery. However, given spinal cord stimulation is invasive and expensive there should be a focus on emerging non-invasive, inexpensive neuromodulation techniques. One such technique (vestibular neuromodulation) being examined by this author: (i) shows promising preliminary clinical results in modulating pain and allodynia; (ii) activates PP-relevant cortical regions (ACC, AIC, S2)^3^; and (iii) modulates belief, cognition, and psychiatric dysfunction (Miller and Ngo, 2007; Ngo et al., 2015; Miller, 2016; Ngo et al., in preparation). Whatever procedural interventions are required, if any, the OPM MoC principles apply throughout planning for and after such interventions.

An OPM MoC has been proposed to optimize PP management and prevention, particularly in compensable environments. It relies heavily upon, but also advances, the comprehensive MoC outlined by Beales et al. (2016) and suggests further strategies for achieving optimal outcomes for injured individuals. Critical to both MoCs is a focus on contemporary pain neuroscience and RTW. The OPM MoC sketched here can be developed and its implementation and efficacy scientifically evaluated (Speerin et al., 2014).

## Author Contributions

The author confirms being the sole contributor of this work and has approved it for publication.

### Conflict of Interest Statement

SM runs a medical consultancy service entitled Occupational Pain Medicine. He also conducts remunerated consultancy work on the clinical panels of WorkSafe Victoria and the Transport Accident Commission, Victoria. The views espoused here are not necessarily the views of these organizations.

## Footnotes

^1^So as not to set potentially unrealistic expectations and thus create further treatment failure and negative context, perhaps the goal of living well with *less* pain is an appropriate compromise.

^2^Most Pain Medicine practices offer biopsychosocial approaches to some extent but the degree to which contemporary pain neuroscience is taught within these varies widely. Moreover, many Pain Medicine practices remain with a strong biomedical focus, searching for specific diagnoses and attempting to eliminate pain generators using one procedural intervention after another.

^3^Interestingly, mechanisms by which spinal cord stimulation reduces pain are also thought to involve the ACC (De Ridder and Vanneste, 2016).
